# A Case of Ovariotomy

**Published:** 1866-02

**Authors:** E. B. Wolcott

**Affiliations:** Milwaukee, Wis.


### CHICAGO MEDICAL EXAMINER.

N. S. DAVIS, M.D., EDITOR.

VOL. VII. FEBRUARY, 1866. NO. 2.

### Original Contributions.

### ARTICLE III.

### A CASE OF OVARIOTOMY.

BY E. B. WOLCOTT, M.D., of Milwaukee, Wis.

EDITOR CHICAGO MEDICAL EXAMINER:

DEAR SIR:—The following case of ovariotomy, as an element of statistics, may be worth reporting. If you think so, please give it a place in the EXAMINER:—

Miss W., aged about 27, some three years and a-half prior to the operation, felt the first symptoms that ultimately called for ovariotomy. In the fall of 1863, one year after the tumor was first discovered, her menses ceased. Soon after, she began to "bloat," as she termed it, and got weak. In May, of 1864, the abdomen filled rapidly with water, and to such an extent that tapping became indispensable. From that time till I first saw her, which was on the 22d of September, 1865, the operation had been repeated nineteen times. I found the abdomen much distended, although but ten days had elapsed since the last tapping. She was failing rapidly in flesh and strength, and presented a clear case for an operation, if, after emptying the abdomen of water, no insuperable difficulties were discovered.

The next day, accompanied by my brother and Dr. SOLON MARKS, I drew off the water, and proceeded to an examination of the tumor, which was favored by the relaxed condition of the abdominal parietes. Although pretty large, no extensive adhesions existed, nor other conditions forbidding an operation, which was, therefore, recommended and without hesitation submitted to.

The incision was made in the usual place and manner—at first, about five inches in length, intending, if, as I suspected, the tumor proved to be multilocular, to reduce the size by emptying the cysts. This was impracticable, from the thick consistence of the contents. I therefore extended the incision to the umbilicus; this enabled me, by pretty firm pressure, to bring the tumor to the surface, through the opening. All adhesions were easily broken by the fingers. I found it attached to the right ovary by a pedicle about two inches wide, by three-quarters of an inch in thickness. This was ligated by passing a double cord through the centre and tying both ways, with great firmness. After detaching the tumor and cleansing the parts in the usual way, the wound was closed by interrupted sutures, including all the structures, save the peritoneum. The ligatures of the pedicle, preserving both ends of each, were twisted into a cord and brought to the surface through the lower angle of the wound, without attempting to confine the pedicle to the surface, which, from its shortness, would have drawn the point of attachment much below the level of the pubes. This disposition of the ligatures was intended to preserve an opening for any matter that might accumulate about the extremity of the pedicle. No adhesive straps were used nor dressing of any kind, save a pledget wet in warm water covering the wound, and changed as often as it became soiled. The patient was now removed to bed, and a grain of morphine administered.

The tumor weighed fifteen pounds; was multilocular, the cysts being filled with a thick, ropy substance.

A brief general outline of subsequent management is all that can be profitable; the details must necessarily vary with each individual case. One of the following pills was given every six hours, beginning in the morning after the operation, which was S ptember 24th:—

℞. Digitalis (leaves,) Quinine, āā ---------- ℈j.

Ext. Hyoscyamus, ---------------------- ℈ij.

Calomel, Ipecac., āā ------------------- gr.v.

Mix for pills No. xx.

The pills were continued for a week, as above, after which they were given once in eight hours. In the meantime occasional doses of morphine and quinine were given when indicated. Bowels were moved on the sixth day after operation, by injection containing emulsion of oil of turpentine, there being some tympanitis. The turpentine was also applied externally over the abdomen, and a bandage snugly pinned over all. Pulse ranged from 90 to 120. Mild, nourishing diet from the first, and on several occasions, where nausea existed, brandy toddy was used.

During the second week, the stitches from the upper portion of the wound were removed.

At the end of the second week, observing an occasional intermission of the pulse, and slight mental disturbance, the pill was given but twice a-day, attributing to the digitalis the above symptoms, and towards the end of the third week discontinued them entirely. The pulse, during this period, ranging from 80 to 100.

On the twentieth day after the operation, a free discharge of matter, somewhat offensive, burst out along the ligatures. This continued, gradually diminishing, about a week after the ligatures came away, which was on the thirtieth day after the operation.

Alternating with the pill, after reducing the number to two a-day, the following prescription was used, of which a teaspoonful was given twice a-day, until after the pill was discontinued, then three times daily for some ten days:—

℞. Quinine, ------------------------------ ʒss.

Elix. Vit., ---------------------------- ʒj.

Tinct. Cantharides, -------------------- ʒj.

Syrup Aurant, -------------------------- ℥iij.

Mix.

In addition to the above, from time to time, as occasion required, an opiate was given, and appropriate remedies to check a disposition to diarrhœa.

The specific effects of the digitalis were observable about a week—they would have been alarming had not the case been known.

In the management of the case, the object was to sustain the vital forces as far as could be done prudently, believing that to be the most successful way to prevent even inflammation and its consequences.

Miss W. is now attending to the domestic affairs of her father's household, in apparently sound health.

